# A case of GLI1-altered mesenchymal pleural tumour with novel gene fusion: a clinical perspective

**DOI:** 10.1093/jscr/rjaf781

**Published:** 2025-09-30

**Authors:** Sheikh Izzat Bin Zainal-Abidin Bahajjaj, Cynthia Ming Li Chia, Benjamin Livingston Farah

**Affiliations:** Department of Cardiothoracic Surgery, Singapore General Hospital, 5 Hospital Drive, Singapore 169609; Department of Cardiothoracic Surgery, Singapore General Hospital, 5 Hospital Drive, Singapore 169609; Department of Anatomical Pathology, Singapore General Hospital, 20 College Road, Singapore 169856

**Keywords:** GLI1, pleural tumour, mesenchymal tumour, gene fusion, NCOR2

## Abstract

This report presents a case of a Glioma-associated homologue-1 (GLI1)-altered mesenchymal tumour with novel gene fusion arising from the pleura in a 25-year-old female undergoing treatment for primary colorectal adenocarcinoma. A pleural nodule was incidentally detected during staging, and biopsy revealed a mesenchymal tumour with a novel NCOR2(exon 7)::GLI1(exon 6) gene fusion. The tumour showed an indolent course over 10 months of surveillance during chemotherapy, with no significant growth. Despite the absence of malignant features, such as high mitotic rate, necrosis, or large size, and a low proliferation index, surgical excision was chosen due to the rarity and uncertain prognosis of this fusion. Histology confirmed a low-grade tumour with unusual SOX10 expression. This case expands the understanding of GLI1-altered mesenchymal tumours, especially in uncommon sites like the pleura, and highlights the importance of multidisciplinary decision-making. Ongoing molecular and pathological analysis is critical to establish robust diagnostic and prognostic frameworks for such rare tumour entities.

## Introduction

Glioma-associated homologue-1 (GLI1)-altered mesenchymal tumours are defined gene fusions or amplification of the GLI1 gene [1]. The GLI1 gene plays a role in pathways that are important for regulating cell differentiation, growth, tissue repair, and regeneration [2]. Tumours found to have this gene amplifications or fusions may occur in various organs and demonstrate a wide biological spectrum [3]. Some show benign behaviour; however, they also have been reported to have locally aggressive behaviour or distant metastases [4]. The pleura itself is a serous mesothelial membrane with a visceral layer and a parietal layer, and primary tumours of the pleura itself are extremely rare [5]. We discuss the clinical perspective of a 25-year-old patient with a GLI1-altered mesenchymal tumour of the pleura. An abstract focusing on the pathological aspects of this case was presented at the Pathology Update 2025 conference in February.

## Case report

**Figure 1:**
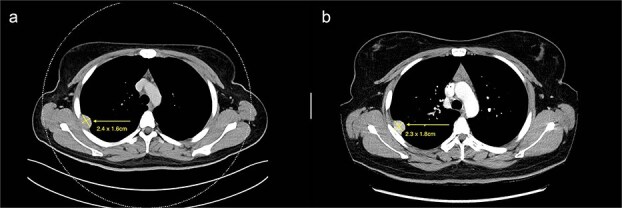
(a) Axial cut of CT thorax taken in April 2024, depicting a right sided hyperdense peripheral pleural lesion measuring 2.4 × 1.6 cm. (b) Axial cut of a repeat CT thorax taken in February 2025, showing the same hyperdense pleural lesion relatively stable in size, now measuring 2.3 × 1.8 cm.

We present a case of a 25-year-old female with a known primary adenocarcinoma of the ascending colon. During staging CT imaging in April 2024, she was found to have a solitary, well-circumscribed right upper lobe pleural nodule (2.4 × 2.1 cm) (Fig. 1a). A CT-guided core biopsy was performed to differentiate between metastatic disease and a second primary lesion. Histopathological analysis revealed a mesenchymal tumour harbouring a novel NCOR2(exon 7)::GLI1(exon 6) gene fusion. This particular fusion has not been previously documented in the literature, making it a noteworthy diagnostic finding.

From a clinical perspective, given the uncertain malignant potential of GLI1 fusion-associated tumours, and in the context of her ongoing adjuvant chemotherapy (8 cycles of XELOX for 6 months) for colorectal adenocarcinoma, surgical management of the pleural lesion was deferred. Serial imaging over 10 months demonstrated tumour stability in size (2.3 × 1.8 cm), suggesting indolent behaviour (Fig. 1b). However, in view of the histological ambiguity and lack of established prognostic markers, despite the seemingly benign behaviour, a shared decision was made to proceed with surgical excision.

**Figure 2:**
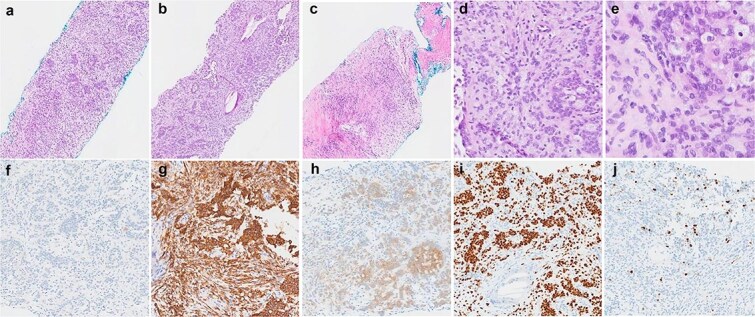
Histological findings of the tumour. (a–c) Haematoxylin and eosin stained sections demonstrating the variety of architectural patterns in the tumour (50× magnification). (d and e) Higher-power photomicrographs demonstrating cellular morphology and cells with clear cytoplasm (D-200× magnification, E-400× magnification). (f–j) Immunoperoxidase stained sections with antibodies for AE1/3 (f), CD56 (g), S100 (h), SOX10 (i), and Ki67 (j) (100× magnification).

The patient underwent a right video-assisted thoracoscopic surgery excision in April 2025. Intraoperatively, a 3 cm pleural lesion was excised without complication, with the gross tumour measuring 3 × 2 × 1.8 cm. Histologically, the tumour consisted of round to ovoid cells arranged in nests, trabeculae, and occasional reticular patterns (Fig. 2). Immunohistochemistry showed strong positivity for S100, CD56, and SOX10, with patchy SMA and CD10 expression. Notably, SOX10 expression is atypical for GLI1-altered mesenchymal tumours. The Ki-67 proliferation index was low (~1%), with no tumour necrosis or increased mitotic activity. The lesion also was noted to retain nuclear staining for PMS2 and MLH1, and is less likely to be related to any possible Lynch Syndrome, given that the patient also has a PMS2 deficient primary colorectal adenocarcinoma.

## Discussion

This case illustrates both diagnostic and clinical management challenges. In general GLI-1 tumours are a new emerging entity, its histological spectrum is not fully defined yet, and there is uncertainty regarding the features that makes the tumour malignant or benign. The NCOR2::GLI gene fusion present here is previously undescribed, the literature currently available generally emphasizes that SOX 10 is negative in these tumours, whereas this particular tumour was SOX 10 positive [3, 6, 7]. There is currently no criteria for malignancy that has been established; however, factors such as tumour necrosis and mitoses **≥**5/10 high power fields tumour size **≥**6 cm and gene rearrangement were proposed, which was not seen in this case [8, 9].

As the pleural nodule remained stable in size between the duration of the interval CT scans that were done, there is clinical reason to believe that the tumour itself was less likely to have locally aggressive behaviour. A case of GLI1-altered mesenchymal tumour with NCOR2(exon 1)::GLI1(exon 4) fusion has been reported, which showed no recurrent disease at 4 months follow-up [3]. We also recognize that the patient was undergoing adjuvant chemotherapy (8 cycles of XELOX for 6 months) during this period that would confound this. However, it is worth noting that XELOX is typically known to be effective in gastrointestinal malignancies, not mesenchymal tumours; hence, it likely had little therapeutic impact [10]. This case also highlights the importance of multidisciplinary decision-making in managing rare tumours with unclear biological potential.

Furthermore from a clinical perspective, while the lesion appeared clinically indolent, the lack of validated criteria for malignancy and the presence of a rare fusion warranted an excisional biopsy. Especially in this patient with no severe medical comorbidities, surgical management can be justified due to diagnostic uncertainty, and given the fact that the general anaesthetic and surgical risks were low in this patient.

## Conclusion

We report a rare case of a pleural-based GLI1-altered mesenchymal tumour with a previously undescribed NCOR2::GLI1 gene fusion and unusual SOX10 positivity in a young adult with synchronous colorectal adenocarcinoma. This case contributes to the growing body of literature on GLI1-altered neoplasms, emphasising the need for further molecular and clinical characterisation to refine prognostic tools that would aid in clinical decision making.
